# A Comparison of 2 Paclitaxel-Coated Balloon Systems in Treatment of De Novo Coronary Artery Lesions

**DOI:** 10.1016/j.jscai.2024.101295

**Published:** 2024-02-15

**Authors:** Alan Yean Yip Fong, Asri Said, Yen Yee Oon, Keng Tat Koh, Kian Hui Ho, Francis Eng Pbeng Shu, Chen Ting Tan, Chandan Deepak Bhavnani, Shaun Wen Huey Lee, Kien Ting Liu, Yee Ling Cham, Tiong Kiam Ong

**Affiliations:** a Department of Cardiology, Sarawak Heart Centre, Ministry of Health Malaysia, Kota Samarahan, Malaysia; b Clinical Research Centre, Institute for Clinical Research, Sarawak General Hospital, Ministry of Health Malaysia, Kuching, Malaysia; c Faculty of Medicine and Health Sciences, Universiti Malaysia Sarawak, Kota Samarahan, Malaysia; d School of Pharmacy, Monash University, Selangor, Malaysia; e National Heart Association of Malaysia, Kuala Lumpur, Malaysia

**Keywords:** de novo lesion, drug-coated balloon, efficacy, mortality, safety

## Abstract

**Background:**

In percutaneous coronary intervention (PCI) of de novo lesions, drug-coated balloons (DCB) have been shown to be a promising strategy to improve clinical outcomes of patients with small vessel disease. Evidence of this strategy in PCI of de novo coronary lesions in a real-world setting is limited. The objective of this study was to compare the 12-month outcomes of 2 paclitaxel-coated balloon systems for the treatment of all de novo coronary artery lesions.

**Methods:**

All patients who were treated for de novo coronary artery stenosis with either SeQuent Please or In.Pact Falcon DCB at a single center from January 2014 to December 2018 were included. The primary end point was the composite of cardiac death, nonfatal myocardial infarction, and target vessel revascularization (3-point major adverse cardiovascular events) at 12 months.

**Results:**

A total of 496 patients with 623 lesions, of which 144 were treated with SeQuent Please and 352 were treated with In.Pact Falcon were included in the study. Baseline patient, lesion and procedural characteristics at baseline were similar between groups. At 12-month follow-up, 3-point major adverse cardiovascular event outcomes were similar (4.2% vs 2.3% respectively; *P* = .272). Deaths due to cardiovascular events were few and similar between groups (2.7% vs 1.1% respectively; *P* = .20).

**Conclusions:**

Both paclitaxel DCB systems have similar efficacy and safety outcomes, suggesting that both may be an appropriate treatment choice for patients with de novo lesions. However, a larger randomized controlled study is needed to confirm these findings.

## Introduction

Coronary artery disease (CAD) is one of the leading causes of morbidity and mortality globally.^1^ Contemporary treatment strategies for severe CAD include percutaneous coronary intervention (PCI) with metallic stent(s), especially for those with acute coronary syndrome (ACS). While effective, in-stent restenosis (ISR) remains a major challenge after stent implantation, especially with the use of bare metal stents (BMS). To address this, drug-eluting stents (DES) were developed, as they allowed for local delivery of antiproliferative agents directly into the coronary artery vessel wall, attenuating neointimal formation which reduced the incidence of ISR.^2^, ^3^, ^4^, ^5^, ^6^, ^7^ However, these stents are associated with the risk of stent thrombosis.^8^^,^^9^

Drug-coated balloons (DCB) were initially developed to treat ISR associated with BMS^10^ and subsequently, those associated with DES.^11^ DCB also allowed for the local delivery of antiproliferative agents directly into the artery wall but without the need for a metallic implant.^12^^,^^13^ This effectively allowed for the rapid and uniform release of antiproliferative drugs throughout the lesion, thus inhibiting the neointimal hyperplasia.^14^ Results from several trials to date have established the role of DCB in the treatment of ISR,^15^, ^16^, ^17^ and has been endorsed in the recent European Society of Cardiology guidelines.^8^

Most of the current DCB approved for clinical application use paclitaxel due to its lipophilic properties, which ensure rapid cellular uptake and a homogenous distribution, allowing for a prolonged effect on the smooth muscle cells.^9^^,^^18^ However, the choice of excipients varies and includes iopromide, urea, dextran or shellac.^19^ These excipients function to facilitate drug retention on the balloon during transit, provide adhesion of the drug to the vessel and promote drug deposition in the tissue.^20^ However, one cannot assume a class effect for all DCB, despite the use of the same cytostatic drug to inhibit restenosis. For example, the SeQuent Please (B. Braun) is a DCB catheter system where paclitaxel is embedded using a hydrophilic iopromide as a carrier.^19^ In comparison, In.Pact Falcon (Medtronic) DCB catheter system uses a proprietary coating with FreePac urea.^21^ Studies to date have suggested that there were different transfer rate patterns of paclitaxel due to the differences in solubility of these carriers.^22^, ^23^, ^24^

Comparison of several DCB to date have suggested discrepancies in the restenosis rates between products, with only 1 study comparing different DCB for de novo lesions.^16^ Indeed, this was recently highlighted by the Asia-Pacific Consensus Group that more research comparing different DCB are needed.^25^ In this study, we present a retrospective analysis of the effects of 2 commonly used paclitaxel-based DCB systems to determine the efficacy in a real-world clinical practice setting in an Asian population, which has not been done before.

## Methods

### Study population

The study was a retrospective analysis of prospectively collected data from the Sarawak Heart Centre, a public access tertiary referral center in Malaysia. Between January 2014 and December 2018, all patients undergoing percutanous coronary intervention (PCI) for CAD were enrolled into the National Cardiovascular Disease–Percutaneous Coronary Intervention Registry. Patients who underwent PCI with DCB (In.Pact Falcon or SeQuent Please) for a de novo coronary artery stenosis were included in this study. Patients with PCI using different DCB during the same setting, involving PCI with a DES in another segment during the same setting, previous PCI, elective surgery within the past 6 months, or intolerant to paclitaxel were excluded from this study.

### PCI

All procedures were performed by a group of experienced interventional cardiologists (experience, 4-20 years) at the hospital, utilizing the same PCI technique. Prior to PCI, antiplatelet treatment was prescribed according to their clinical presentation, including unfractionated heparin (between 70-100 UI/kg body weight). Glycoprotein IIb/IIIa inhibitor tirofiban was administered at operator’s discretion. The target coronary lesion was prepared with an appropriately sized noncompliant balloon (1:1 ratio to the size of the reference vessel). Plaque modification using a scoring balloon or rotational atherectomy was occasionally used, at the discretion of the operator. Following preparation of the lesion, DCB would be deployed if there was <30% residual stenosis, only minor dissection, and TIMI 3 flow. The DCB was sized to the reference vessel diameter, and the length of at least the segment of the target lesion identified at the start of the procedure. Patients were treated using the choice of balloon of either SeQuent Please DCB or In.Pact Falcon DCB based upon the discretion of the attending cardiologist. Both DCB are coated with 3 μg/mm^2^ of paclitaxel, with the former using iopromide as the hydrophilic excipient while the latter using urea as the inert excipient. Additional DES was implanted in the event of significant dissection (NHLBI Classification Type C, D, or E intimal tears, or TIMI <3 flow—a technique known as “bailout stenting” as recommended by the Asia-Pacific Consensus Group for DCB treatment of CAD).^25^

### Follow-up

Data were collected using electronic form during follow-up at 1, 3, 6, and 12 months. Procedural success was defined as device success without the occurrence of death, myocardial infarction (MI), or repeat revascularization of the target lesion during the hospital stay. Cause of death was determined from the National Death Register, and where possible, confirmed with local medical records.

### Primary outcome

The primary end point of interest was the composite of cardiac death, nonfatal MI, and target vessel revascularization (defined as “3-point major adverse cardiovascular events [MACE]”) at 12 months. Cardiac death was defined as any death that was not clearly of extracardiac origin or due to MI. Secondary outcomes of interest were the rates of cardiac death, target lesion thrombosis, need for bailout stenting and dissection and hospitalization days.

### Statistical analysis

Data are expressed as mean with SD or frequency with corresponding percentage. To compare the difference in end points, we used the Pearson χ^2^ and Fisher exact tests for categorical analysis and independent *t* test or Mann-Whitney *U* test for numerical analysis. Clinical events were calculated using Kaplan-Meier analysis, with between-group comparison using log-rank test. The hazard ratio (HR) and 95% CI were calculated using the Cox proportional hazard model, assuming proportional hazards based upon Schoenfeld residual. A 2-sided *P* < .05 was used as a cut-off. All statistical analyses were performed using Stata version 16 (StataCorp LLC). The study was approved by the Malaysian Research Ethics Committee (NMRR-07-20-250).

## Results

During the study period, a total of 4693 patients underwent PCI at Sarawak Heart Centre, of which 496 patients with de novo lesions were treated with a DCB (Supplemental Figure S1). This comprised 144 procedures using the SeQuent Please DCB and 352 using the In.Pact Falcon DCB system. Baseline clinical and angiographic characteristics were similar between both groups (Table 1). The median age of participants was 58.4 ± 16.2 years, 82.9% of them were males, and 26.8% were smokers. Comorbidities were common, of which 70.6% had hypertension, 59.7% had dyslipidemia and 36.7% had type II diabetes. In addition, 34.1% had a previous MI. Baseline participant characteristics or lesion characteristics did not differ between both DCB groups or with patients who sought care for PCI at the center since 2013 (Tables 1 and 2 and Supplemental Figure S2).

**Table 1:** Demographic characteristics of participants included in the current study.

	SeQuent Please DCB (n = 144)	In.Pact Falcon DCB (n = 352)	Patients with DES at the center from 2014-2018 (n = 1520)
Clinical characteristics			
Age, y	56.6 (17.1)	58.8 (15.9)	57.2 (10.6)
Female sex	20 (13.9)	65 (18.5)	206 (13.6)
Current smoker	41 (28.5)	92 (26.1)	548 (36.1)
Body mass index, kg/m^2^	26.9 ± 5.1	26.5 ± 4.3	26.3 ± 4.4
Coronary artery disease at presentation			
STEMI	45 (31.3)	80 (22.7)	748 (49.1)
NSTEMI	26 (18.1)	64 (18.2)	372 (24.5)
Unstable angina	13 (9.0)	36 (10.2)	175 (11.5)
Chronic stable angina	60 (41.7)	172 (48.9)	225 (14.8)
Comorbidities			
Type 2 diabetes	59 (41.0)	123 (34.9)	459 (30.2)
Dyslipidemia	79 (54.9)	217 (61.6)	733 (48.2)
Heart failure	17 (11.8)	42 (11.9)	110 (7.2)
Hypertension	100 (69.4)	250 (71.0)	911 (59.9)
Previous myocardial infarction	58 (40.3)	111 (31.5)	663 (43.6)
Coronary artery disease^b^	43 (29.9)	125 (35.5)	394 (25.9)
Peripheral vascular disease	3 (2.1)	3 (0.9)	8 (0.5)
Chronic kidney disease	6 (4.2)	13 (3.7)	52 (3.4)
Killip class			
1	97 (67.4)	226 (64.2)	1079 (71.0)
2	4 (2.8)	18 (5.1)	74 (4.9)
3	0 (0.0)	2 (0.6)	9 (0.6)
4	4 (2.8)	3 (0.9)	63 (4.2)
Angiographic characteristics			
Severe calcification	12 (8.3)	22 (6.2)	86 (5.9)
Mean diameter of DCB, mm	2.63 ± 0.41	2.61 ± 0.46	2.99 ± 0.46
Maximal inflation pressure, atm^a^	12.67 (3.54)	11.27 (3.97)	16.94 (3.28)

Data are presented as median (IQR), frequency (%), or mean ± SD. The Mann-Whitney *U* test or χ^2^ test was used for between-group comparisons, as appropriate.

DCB, drug-coated balloon; DES, drug-eluting stent; STEMI, ST-elevation myocardial infarction; NSTEMI, non-ST-elevation myocardial infarction.

a Data only available from 2017 onward.

b Reported presence of >50% stenosis on CT angiography, angiogram, or ischemia on functional cardiac imaging such as nuclear, MRI, and echo. A positive treadmill test or high calcium score alone is not sufficient.

**Table 2:** Lesion characteristics and procedural data.

Variables	Total lesions (n = 623)	SeQuent Please DCB (n = 180)	In.Pact Falcon DCB (n = 443)	*P* value
Number of patients	496	144	352	
Angiographic findings^a^				.726
Single-vessel disease	388 (62.3)	111 (61.7)	277 (62.5)	
Multivessel disease	223 (35.8)	64 (35.6)	159 (35.9)	
Left main/left main stem	5 (0.8)	2 (1.1)	3 (0.7)	
Graft	7 (1.1)	3 (1.7)	4 (0.9)	
Location of lesion^a^				
Left anterior descending artery	346 (55.5)	105 (58.3)	241 (54.4)	.353
Left circumflex artery	157 (25.2)	49 (27.2)	108 (24.4)	
Right coronary artery	111 (17.8)	25 (13.9)	86 (19.4)	
Left main stem	5 (0.8)	0 (0.0)	4 (0.9)	
Graft	4 (0.6)	1 (0.6)	4 (0.9)	
Severe calcification	34 (6.9)	14 (8.4)	25 (6.0)	.278
Lesion and device characteristics				
Estimated lesion length, mm	20.0 (15.0)	26.0 (17.0)	18.0 (17.0)	<.001
DCB length, mm	25.0 ± 12.0	29.9 ± 12.0	26.7 ± 13.7	<.001
DCB diameter, mm	2.5 (0.5)	2.8 (0.5)	2.5 (0.5)	.027
Predilatation balloon diameter, mm	2.45 ± 0.43	2.44 ± 0.43	2.45 ± 0.44	.775
DCB deployment pressure, atm	11.76 ± 4.30	11.55 ± 4.10	11.81 ± 4.35	.684
Complications				
Dissection^a^	41 (6.6)	13 (6.9)	28 (6.3)	.999
Flow limiting	1 (0.2)	0 (0)	1 (0.2)	
Nonflow limiting	40 (6.4)	13 (6.9)	27 (6.1)	
Bailout stenting^a^	15 (2.4)	6 (3.33)	9 (2.0)	.388

Data are presented as median (IQR) or frequency (%). The Mann-Whitney *U* test or *t* test was used for between-group comparisons, as appropriate.

DCB, drug-coated balloon.

*P* values based upon *t* test or Mann-Whitney *U* test unless otherwise stated.

a *P* value based upon χ^2^ test or Fisher exact test.

### Procedural and lesion characteristics

A total of 623 lesions were treated with a median lesion length of 20.0 mm (15.0), with most presenting with single vessel disease (62.3%). The left anterior descending artery was the most frequent target vessel for DCB intervention in both groups (58% vs 54%; *P* = .35). Mean length of stay was similar between SeQuent Please DCB and In.Pact Falcon DCB arm (3.00 vs 2.00 days; *P* = .61). No differences between both groups were noted in the inflation pressure of the predilatation balloon and the extent of coronary disease. However, patients in the In.Pact Falcon DCB arm were treated with shorter and smaller diameter DCB compared to the SeQuent Please DCB arm. Bailout stenting was required in 2.4% of the cases (Table 2).

### Efficacy outcome

**Figure 1:**
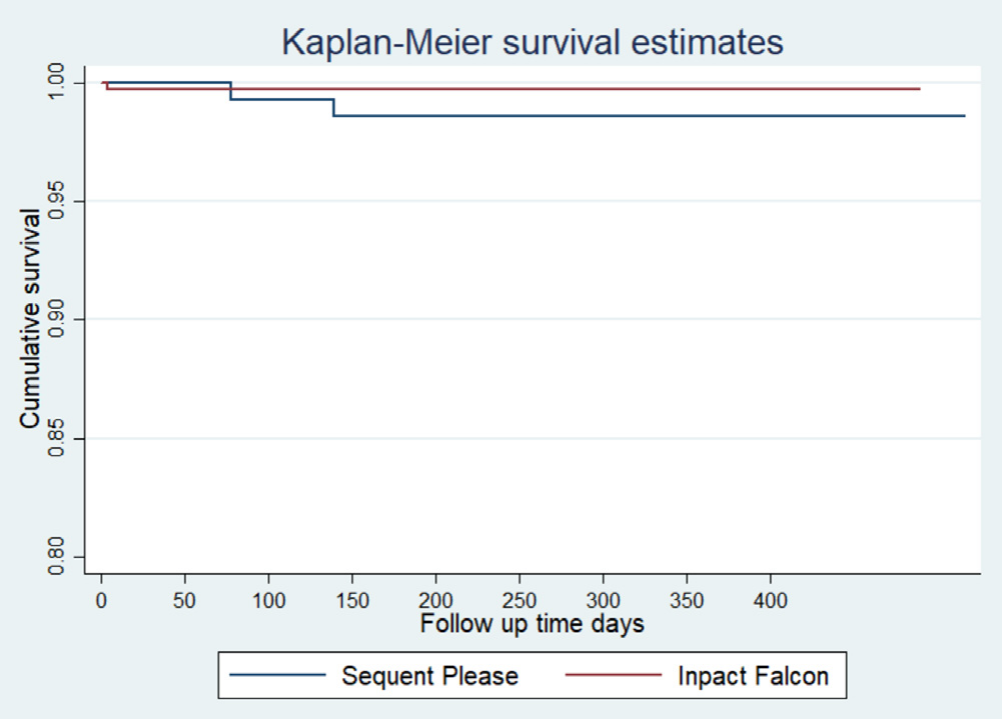
**Kaplan-Meier survival curves for MACE outcomes at 12 months.** MACE, major adverse cardiovascular events.

Complete follow-up data were available at 1 year for 488 (98.4%) patients, with 144 patients in the SeQuent Please DCB arm and 344 patients in the In.Pact Falcon DCB arm (Table 3, Figure 1). The primary outcome was similar in both the SeQuent Please DCB and In.Pact Falcon DCB arms (4.2% vs 2.3%; *P* = .272). All-cause mortality rate after 12 months postprocedure was 5.4%, with relatively similar rates in both SeQuent Please DCB and In.Pact Falcon DCB arms (8.3% vs 4.0%; HR, 0.48; 95% CI, 0.23-1.01). Cardiovascular mortality was similar, with 4 patients (2.7%) in the SeQuent Please DCB arm and 4 patients (1.1%) in the In.Pact Falcon DCB arm (HR, 0.41; 95% CI, 0.10-1.61). There were 7 cases of target lesion thrombosis, 1 (0.7%) in the SeQuent Please arm and 6 (2.7%) in the In.Pact Falcon arm (HR, 0.40; 95% CI, 0.05-3.29).

**Table 3:** Key clinical and safety outcomes of the study.

Outcome	SeQuent Please DCB (n = 144)	In.Pact Falcon DCB (n = 344)	Hazard ratio (95% CI)	*P* value
Primary outcome				
3-point MACE—cardiac death, nonfatal MI, and target vessel revascularization	6 (4.2)	8 (2.3)	0.55 (0.19-1.61)	.272
Safety outcomes				
12-month all-cause death	12 (8.3)	14 (4.0)	0.48 (0.23-1.01)	.059
Cardiac death	4 (2.7)	4 (1.1)	0.41 (0.10-1.61)	.215
Noncardiac death	6 (5.5)	5 (1.4)	2.87 (0.89-9.24)	.078
Target lesion thrombosis	1 (0.7)	6 (1.7)	0.40 (0.05-3.29)	.391
Dissection	13 (6.9)	28 (6.3)	1.11 (0.59-2.08)	.323
Bailout stenting	6 (3.33)	9 (2.0)	1.59 (0.57-4.39)	.369
Hospitalization, d	3.0 (3.0)	2.0 (3.0)	–	.613

Data are presented as frequency (%) or median (IQR).

DCB, drug-coated balloon; DES, drug-eluting stent; MACE, major adverse cardiovascular events; MI, myocardial infarction.

## Discussion

Most randomized controlled study data to date on de novo lesions have compared DCB with DES.^10^^,^^11^^,^^26^^,^^27^ In particular, these are often the iopromide-based SeQuent Please DCB, with more limited data available for the urea-based In.Pact Falcon.^13^ There is no head-to-head comparison of these 2 different DCB systems to date in a real-world clinical setting. In this retrospective analysis of the National Cardiovascular Disease Database of Malaysia, we found that the urea-based DCB was noninferior to the iopromide-based DCB with regard to the primary end point of 12-month all-cause mortality in treating de novo lesions. Our results support the clinical equivalency of both DCB using paclitaxel, and that different drug carriers and coating techniques were safe and feasible for de novo lesions (as summarized in the Central Illustration below).

**Central Illustration:**
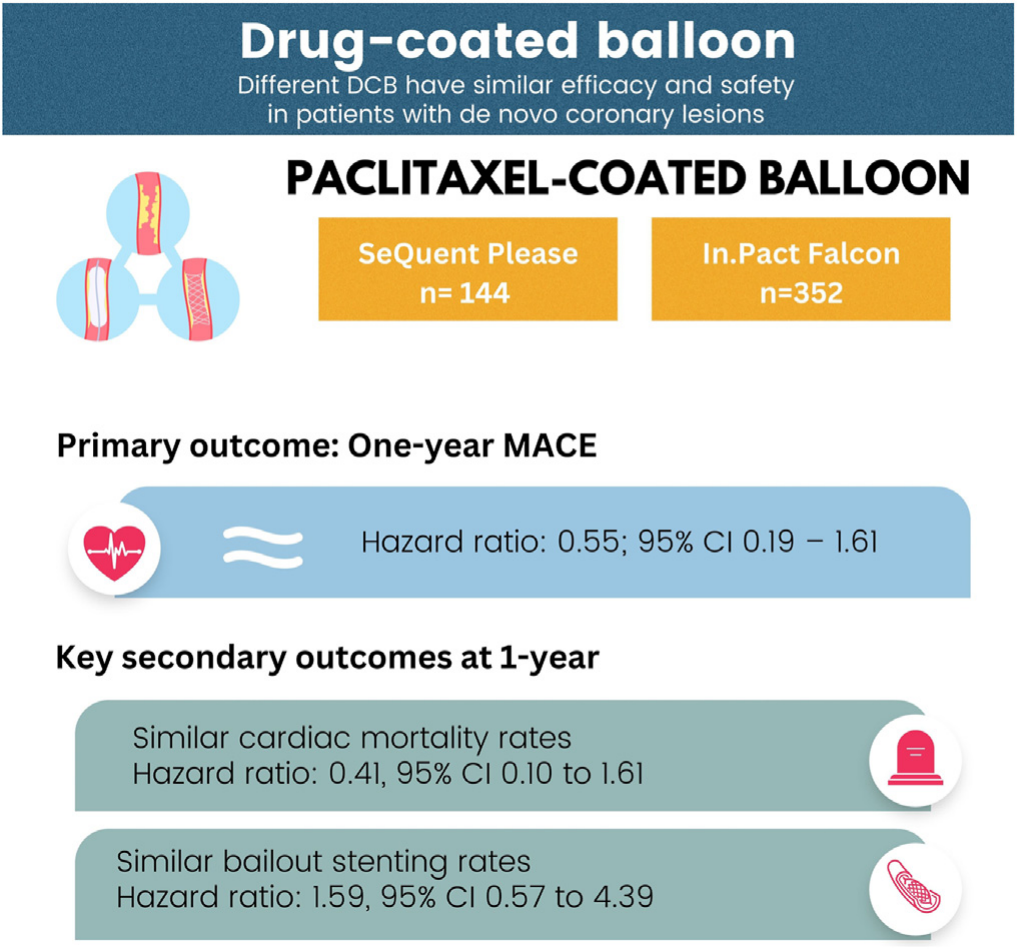
Efficacy and safety of 2 drug-coated balloons for de novo coronary artery lesions.

There are currently multiple modalities for treatment of coronary lesions, including implant-based technologies such as BMS, DES, and more recently implant-free technology such as DCB.^12^ DCB represent an attractive option as they offer the promise of maintaining patency while reducing the need for additional stents. The efficacy of paclitaxel in reducing restenosis has been previously reported with the use of different DCB technologies in various clinical studies and meta-analyses.^13^ The findings from this study concur with previous reports, suggesting limited differences in the efficacy of DCB, despite variations in the antiproliferative drugs, doses (varying from 2 to 3.5 g/mm^2^), excipients used as well as the coating technology.

The PICCOLETO study was stopped prematurely due to a higher rate of MACE after 9 months in the DCB arm.^28^ In comparison, the BELLO study showed superiority of the In.PACT Falcon DCB vs DES, with a lower 3-year MACE of 14% compared to 30% with DES.^26^ Similarly, in the large BASKET SMALL II study, the authors showed that SeQuent Please DCB had comparable MACE rates at 12 months compared to the DES group.^29^ The FALCON registry reported an overall MACE rate of 9.7% at 12 months in a real-world setting.^17^ Similarly, the study by Venetsanos reported comparable 4-year long-term efficacy with 3 currently available DCB brands, including SeQuent Please DCB and In.Pact Falcon DCB, with a similar rate of MACE, clinically driven restenosis and target lesion thrombosis.^16^

Our study has several strengths. Data from this paper are one of the largest comparing SeQuent Please DCB with In.Pact Falcon DCB, with a large number of long-term follow-up (488 at 1 year). This is also 1 of the first few studies to our knowledge which had compared different paclitaxel-based DCB systems in an Asian setting as most other studies were done in Western populations. However, in a real-world study, selection bias and unmeasured confounders cannot be ruled out.

Finally, evidence supporting the use of both these paclitaxel-based DCB systems provides greater impetus toward expanding its use for PCI in de novo lesions, not only for those with diffuse or small caliber coronary lesions. This has implications in Asia, where a significant number of patients treated at tertiary centers are usually located in urban areas, and then return to their community settings in rural areas. Where there may be limited access to regular medications, prolonged antiplatelet therapy interruption following DES replacement would expose the patient to an elevated risk of potentially life-threatening ST. A stent-free strategy, for example, using a DCB approach, could mitigate this. A recent survey of the use of DCB for PCI in the Asia-Pacific region has also indicated greater interest in this field.^30^

### Limitations

This registry analysis has some limitations. All patients included were from a single center and were not randomized. However, in our single-center study, all attending cardiologists used a single technique which minimized operator variability seen in other studies. While DES is the most commonly used intervention for de novo coronary lesions, results of this study showed very similar clinical outcomes, suggesting that DCB could be an equally effective strategy (Supplemental Tables S1 and S2). Bailout stenting was only utilized when there was significant compromise in coronary flow post DCB deployment, with comparable rates to literature.^31^ Late lumen loss, a common variable in PCI outcome assessment was not assessed here, as there was no routine follow-up angiography performed in our usual clinical practice. Nonetheless, this study was aimed primarily at assessing clinical outcomes at 1 year. Finally, the overall efficacy reported in our study may not be applicable to other PCI centers with limited experience in the use of DCB in treating de novo lesions or utilizing different PCI techniques other than that documented here.

## Conclusion

This registry-based study showed the equivalence of 2 different paclitaxel DCB systems for PCI of de novo lesions in an Asian population. DCB may be an attractive alternative to DES for most de novo coronary lesions, particularly in the Asian setting.
