# Supplementary material for: A Comparison of 2 Paclitaxel-Coated Balloon Systems in Treatment of De Novo Coronary Artery Lesions
Source: J Soc Cardiovasc Angiogr Interv. 2024 Feb 15;3(5):101295. doi: 10.1016/j.jscai.2024.101295 (PMC11308760; doi:10.1016/j.jscai.2024.101295)

Supplemental Table S1. Lesion characteristics of participants who underwent percutaneous coronary intervention at Sarawak Heart Centre

Variables	SeQuent Please DCB (n=180)	In.Pact Falcon DCB (n=443)	Drug-eluting stent (n=1696)
**Number of patients**	**144**	**352**	**1,520**
**Angiographic findings**			
Single vessel disease	111 (61.7)	277 (62.5)	1164 (71.9)
Multiple vessel disease	64 (35.6)	159 (35.9)	417 (25.8)
Left main/ Left main stent	2 (1.1)	3 (0.7)	28 (1.7)
Graft	3 (1.7)	4 (0.9)	9 (0.6)
**Location of lesion, n (%)**			
Left anterior descending artery	105 (58.3)	241 (54.4)	1,262 (74.4)
Left circumflex artery	49 (27.2)	108 (24.4)	449 (26.5)
Right coronary artery	25 (13.9)	86 (19.4)	774 (45.6)
Left main stem	0 (0.0)	4 (0.9)	30 (1.77)
Graft	1 (0.6)	4 (0.9)	9 (0.53)
Severe calcification, n (%)	12 (8.3)	22 (6.2)	102 (6.30)
**Lesion and device characteristics**			
Estimated lesion length, mm median (IQR)	26.0 (17.0)	18.0 (17.0)	12.0 (13.0)
DCB length, mean (SD) mm	29.9 (12.0)	26.7 (13.7)	23.5 (8.9)
DCB diameter, median (IQR) mm	2.8 (0.5)	2.5 (0.5)	3.0 (0.5)
Pre-dilatation balloon diameter, mean (SD) mm	2.44 (0.43)	2.45 (0.44)	2.70 (0.56)
DCB Balloon deployment pressure, mean (SD)	11.55 (4.10)	11.81 (4.35)	16.50 (3.74)
**Complications, n (%)**			
Dissection	13 (6.9)	28 (6.3)	29 (1.7)
Flow limiting	0 (0)	1 (0.2)	3 (0.2)
Non-flow limiting	13 (6.9)	27 (6.1)	23 (1.4)

Supplemental Table S2. Clinical outcomes of participants who underwent percutaneous coronary intervention at Sarawak Heart Centre

**Outcome**	**SeQuent Please DCB (n=144)**	**In.Pact Falcon DCB (n=352)**	**Drug-eluting stent (n=1520)**
**12-month all cause death**	12 (8.3)	14 (4.0)	96 (6.3)
Cardiac death	4 (2.7)	4 (1.1)	42 (2.8)
Non-cardiac death	6 (5.5)	5 (1.4)	32 (2.1)
Unknown	2 (1.4)	5 (1.4)	22 (1.5)
Dissection	13 (6.9)	28 (6.3)	15 (1.0)

Supplemental Figure S1. Study flow chart of patients who sought care for percutaneous coronary intervention at Sarawak Heart Centre

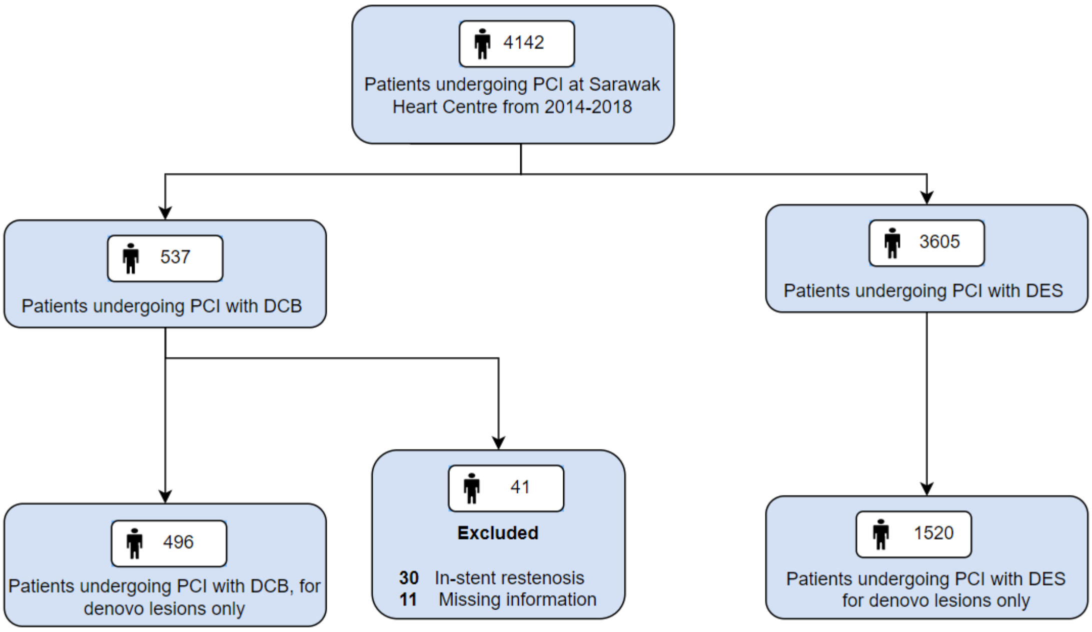

Supplemental Figure S2. Distribution of utilisation of SeQuent Please and In.Pact Falcon for de novo lesions over the study period

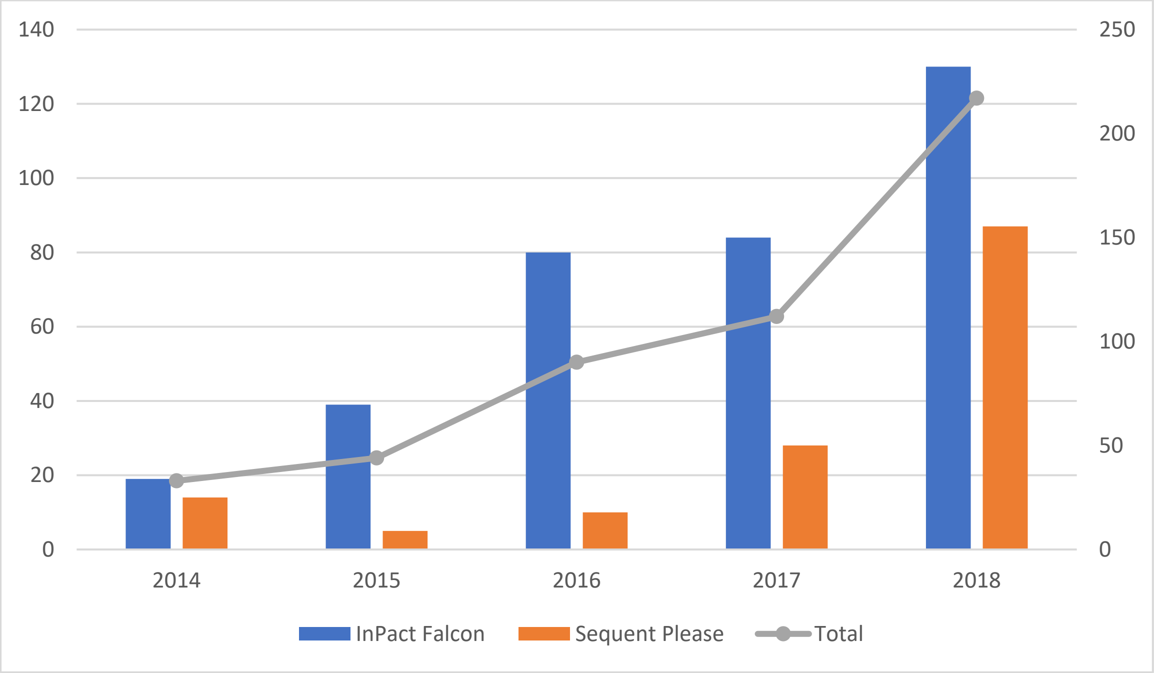

Supplement: Supplemental Figures S1 and S2 and Supplemental Tables S1 and S2 [file mmc1.pdf]
